# Low heritability in pharmacokinetics of talinolol: a pharmacogenetic twin study on the heritability of the pharmacokinetics of talinolol, a putative probe drug of MDR1 and other membrane transporters

**DOI:** 10.1186/s13073-016-0372-2

**Published:** 2016-11-08

**Authors:** Johannes Matthaei, Mladen V. Tzvetkov, Valerie Gal, Cordula Sachse-Seeboth, Daniel Sehrt, Jakob B. Hjelmborg, Ute Hofmann, Matthias Schwab, Reinhold Kerb, Jürgen Brockmöller

**Affiliations:** 1 Institute for Clinical Pharmacology, University Medical Center, Georg-August University, Robert-Koch-Straße 40, 37075 Göttingen, Germany; 2 Department of Epidemiology, Biostatistics and Biodemography, University of Southern Denmark, J. B. Winsløwsvej 9B, 5000 Odense, Denmark; 3 Dr. Margarete Fischer-Bosch Institute of Clinical Pharmacology, University of Tübingen, Auerbachstraße 112, 70376 Stuttgart, Germany; 4 Department of Clinical Pharmacology, University Hospital Tübingen, Auf der Morgenstelle 8, 72076 Tübingen, Germany; 5 Department of Pharmacy and Biochemistry, University of Tübingen, Auf der Morgenstelle 8, 72076 Tübingen, Germany

**Keywords:** Talinolol, MDR1, P-glycoprotein, ABCB1, MDR5, MRP2, ABCC2, BCRP, Heritability, Twin study

## Abstract

**Background:**

Efflux transporters like MDR1 and MRP2 may modulate the pharmacokinetics of about 50 % of all drugs. It is currently unknown how much of the variation in the activities of important drug membrane transporters like MDR1 or MRP2 is determined by genetic or by environmental factors. In this study we assessed the heritability of the pharmacokinetics of talinolol as a putative probe drug for MDR1 and possibly other membrane transporters.

**Methods:**

Talinolol pharmacokinetics were investigated in a repeated dose study in 42 monozygotic and 13 same-sex dizygotic twin pairs. The oral clearance of talinolol was predefined as the primary parameter. Heritability was analyzed by structural equation modeling and by within- and between-subject variance and talinolol clearance was correlated with polymorphisms in MDR1, MRP2, BCRP, MDR5, OATP1B1, and OCT1.

**Results:**

Talinolol clearance varied approximately ninefold in the studied sample of healthy volunteers. The correlation of clearances between siblings was not significantly different for the monozygotic and dizygotic pairs. All data analyses consistently showed that variation of talinolol pharmacokinetics was mainly determined by environmental effects. Structural equation modeling attributed 53.5 % of the variation of oral clearance to common environmental effects influencing both siblings to the same extent and 46.5 % to unique environmental effects randomly affecting individual subjects. Talinolol pharmacokinetics were significantly dependent on sex, body mass index, total protein consumption, and vegetable consumption.

**Conclusions:**

The twin study revealed that environmental factors explained much more of the variation in pharmacokinetics of talinolol than genetic factors.

**Trial registration:**

European clinical trials database number: EUDRA-CT 2008-006223-31. Registered 26 September 2008. ﻿ClinicalTrials.gov number: NCT01845194.

**Electronic supplementary material:**

The online version of this article (doi:10.1186/s13073-016-0372-2) contains supplementary material, which is available to authorized users.

## Background

Efflux transmembrane transport of drugs and other molecules is highly variable within and between humans due to inherited and environmental factors. This can have profound consequences for the intended and adverse effects of drugs and for the biological effects of many other endogenous and exogenous molecules. Efflux transporters first came into focus as mediators of multi-drug resistance in cancer chemotherapy [1, 2]. Among the efflux transporters, multi-drug resistance protein 1 (MDR1, P-glycoprotein), encoded by the *ABCB1* gene, has been the most extensively studied [3–9]. MDR1 is expressed on the apical membrane of enterocytes, hepatocytes, and renal tubular cells and at the blood–brain barrier and the blood–testes barrier and protects cells and organs against overload with numerous substances [10, 11]. Some animals completely lack MDR1 but we do not know of any humans completely lacking it [10]. Nevertheless, MDR1 expression and activity vary substantially between individuals. MDR1 expression is regulated by several mechanisms and substantial up-regulation can be induced by several substances and is mediated by PXR, RXR, and other nuclear transcription factors [12]. On the other hand, numerous environmental substances and drugs, such as cyclosporine or verapamil, can substantially inhibit P-glycoprotein [13, 14].

Inherited polymorphisms may contribute to variation in MDR1 activity. In particular, the polymorphisms C1236T (silent), G2677T/A (Ala893Ser/Thr), and C3435T (silent) have been extensively studied [15–24]. Understanding of the functional role of these variants in humans is complicated by the fact that these variants are in strong linkage disequilibrium [25, 26]. Genetic polymorphisms may play some role in the variation of MDR1 expression and activity. However, the genotype–phenotype correlation data obtained by different investigators is not entirely consistent. In conclusion, it is currently unknown how much of the variation in MDR1 activity as tested in vivo with putative probe drugs like talinolol or digoxin is explained by environmental or heritable factors.

Another efflux transporter of particular interest for the disposition of drugs, other foreign compounds, and endogenous substrates is the multidrug resistance-associated protein 2 (MRP2; also known as ABCC2 (ATP binding cassette subfamily C member 2)). MRP2 is expressed in a broad range of tissues and also contributes to multi-drug resistance in cancer therapy [27]. It is expressed on the apical membrane of cells in the liver, intestine, and kidneys [28, 29]. MRP2 is an important efflux transporter of conjugated bilirubin and inherited lack of activity is known as Dubin–Johnson syndrome [30]. Furthermore, MRP2-mediated drug interactions may cause liver toxicity [31]. Activity of MRP2 can also be greatly modified by transcriptional regulation and by inhibition of its transport function, but also by genetic polymorphisms. Among others, the impact of two genetic polymorphisms (-C24T and G1249A in *MRP2*) on, for example, the bioavailability of mycophenolate mofetil [32, 33] and tacrolimus [34, 35], have been extensively studied, with controversial results.

A number of probe drugs for the in vivo study of MDR1 activity have been suggested but none of them is perfect [36, 37]. Because of the low substrate specificity of many drug membrane transporters, many probe drugs may reflect the activity of several transporters. For instance, a typical probe drug for MDR1, fexofenadine, is also a substrate of several OATP influx transporters [37]. In this study, talinolol was chosen as a probe drug for drug membrane transporters. Talinolol appears suitable as a membrane transporter probe drug since less than 1 % of an orally administered dose was recovered as metabolites in urine [38, 39]; thus, metabolism plays only a minor role in its elimination.

Evidence for which transporters are possibly relevant in the pharmacokinetics of talinolol is based on multiple data sources. Talinolol is widely considered as an MDR1 probe drug both in vitro and in vivo [36]. Several investigators used talinolol in caco-2 cells as a model substrate of MDR1. Plasma concentrations after oral administration of talinolol were substantially (4.5-fold) higher in a MDR1 knockout rat compared with the wild-type rat [40]. Clinically, net intestinal secretion of talinolol was inhibited by the MDR1 inhibitor verapamil [41] and carbamazepin [42]. Additionally, rifampin-induced MDR1 and MRP2 expression resulted in decreased oral bioavailability of talinolol [43] and corresponding effects were seen with St. John’s wort [44]. Unexpectedly, two inhibitors of MDR1, grapefruit juice and verapamil, decreased oral bioavailability of talinolol, which was explained by preferential inhibition of intestinal influx transporters by polytropic inhibitors like verapamil [45, 46]. According to these data, it is very likely that MDR1 modulates the pharmacokinetics of talinolol in humans to a relevant extent.

MRP2 may also modulate the pharmacokinetics of talinolol. Expression of both MDR1 and MPR2 is greatly increased by typical inducers like carbamazepin and rifampin and the effects of these inducers on talinolol pharmacokinetics may thus also be explained by an involvement of MRP2. This is also supported by the low talinolol oral bioavailability found in carriers of the *MPR2* exon 10 Val417Ile genotype (G1249A) [47].

A comprehensive in vitro screen of all influx and efflux transporters possibly relevant in talinolol pharmacokinetics has not been performed to our knowledge. Preliminary data indicated that talinolol may be a substrate of OATP1B1 [48] and with its cationic structure, talinolol may be a substrate of OCT1. In addition, other efflux transporters, including the polymorphic MDR5 (ABCB5) and BCRP (ABCG2) [49], may modulate the pharmacokinetics of talinolol; therefore, we also explore here the impact of polymorphisms known to be functional in these transporters.

The aim of the present study was to reveal how much of the variation in talinolol pharmacokinetics is due to heritable factors. Secondary questions were whether we can reproduce the effects of genetic polymorphisms which were associated with talinolol pharmacokinetics in earlier publications and how suitable a much lower test dose of talinolol (2.5 mg) might be for in vivo phenotyping. A low dose is apparently more safe and the lower dose was also scientifically interesting because the impact of MDR1 on talinolol pharmacokinetics might depend on the dose [50].

## Methods

### Subjects and study design

Monozygotic (MZ) and same-sex dizygotic (DZ) twin pairs were included if they were healthy according to medical history, clinical examination, electrocardiogram, and standard clinical biochemistry and hematology tests. Except for oral contraceptives no other drugs were allowed for 1 week prior to and 48 h after administration of talinolol.

A single dose of 50 mg talinolol (Cordanum®, AWD.pharma, Dresden, Germany) was administered to 114 subjects. Among these subjects were 43 MZ and 13 DZ twin pairs; two single twins were excluded from the analyses since the second twin could not participate in the study. Additionally, one MZ pair was excluded from the analyses because of missing concentration data for the last blood sample 22 h after talinolol administration. Therefore, all analyses on 50 mg talinolol are based on data from 110 subjects (42 MZ and 13 DZ twin pairs). Per protocol, talinolol 50 mg was administered on three different occasions to each subject with an at least 7-day wash-out period in between. Blood sampling was done prior to and 0.5, 1, 2, 3, 4, 5, 6, and 22 h after talinolol administration. Talinolol was part of a phenotyping drug cocktail in which midazolam, torsemide, metoprolol (all intravenously) [51], and caffeine (orally) [52] were also administered in a sequential order in the first 2 h of each study day. All the substances in this cocktail are not known to have any pharmacokinetic interactions and talinolol was the last administered drug and was given 1 h after caffeine.

In an additional add-on study aiming to compare low-dose talinolol, which is presumed to lack relevant cardiovascular effects, 2.5 mg talinolol was administered to 73 subjects: 29 MZ and 7 DZ twin pairs. One single twin was excluded from the analyses. The capsules containing 2.5 mg of talinolol were prepared from Cordanum® 50 tablets, which were ground and used to refill gelatin capsules in 2.5 mg portions. In this add-on study, blood sampling was done prior to and 0.5, 1, 1.5, 2, 3, 4, 6, and 8 h after talinolol administration.

Blood samples were centrifuged for 10 minutes (4000 rpm, 4 °C) and plasma samples were stored at −20 °C until laboratory analysis.

### Talinolol plasma concentrations

Talinolol plasma concentrations were determined by liquid chromatography–tandem mass spectrometry (LC-MS/MS) analysis using deuterium labeled talinolol-d5 (Toronto Research Chemicals, Nr. T005002) as internal standard. For the 50 mg talinolol study sample preparation with protein precipitation and HPLC separation were performed as described [51]. Samples from the low-dose study were worked up with solid phase extraction. LC-MS/MS analysis was carried out on an Agilent 6460 triple quadrupol mass spectrometer (Agilent, Waldbronn, Germany) coupled to an Agilent 1200 HPLC system. Ionization mode was electrospray (ESI), polarity positive. The mass spectrometer was operated in the multiple reaction monitoring (MRM) mode using the transitions *m/z* 364.3 to 308 and *m/z* 369.3 to 313 for talinolol and the internal standard, respectively. For the 50-mg dose study, the limit of quantification (LOQ) was 1.5 pmol/ml. Quality control samples over the whole concentration range could be determined with a coefficient of variation (CV) better than 2.1 %. For the low-dose study, the LOQ was 0.2 pmol/ml. Over the whole concentration range (from 0.2 to 30 pmol/ml), a CV better than 5 % was achieved.

### Molecular genetic analyses

Genotyping was performed by single-base primer extension using fluorescence-labeled dideoxynucleotides followed by capillary electrophoresis. Genotype calling was performed using Gene Mapper v3.7 Software ® (Applied Biosystems ®, Foster City, CA, USA). The following variants of efflux transporters were genotyped: *MDR1* C1236T (rs1128503), G2677T/A (rs2032582), and C3435T (rs1045642); *MDR5* C392T (rs17143212); *MRP2 *G1249A (rs2273697) and -C24T (rs717620), *BCRP* (*ABCG2*) G34A (rs2231137) and C421A (rs2231142). Additionally, the following haplotypes of the influx transporters were determined: OATP1B1 *1a, *1b, *5, *14, *15, and *OCT1* alleles *1 to *10. In total, variants in 23 polymorphic genes were genotyped for assessment of zygosity. Monozygosity was concluded when both siblings of each pair had completely identical genotypes for all 23 polymorphic loci.

### Statistics

Pharmacokinetic parameters were estimated with WinNonlin (Pharsight Corporation, Mountain View, USA). Total clearance, the primary parameter, normalized to bioavailability (Cl/F) was calculated as dose/AUC_infinity_ and in this study is referred to as “clearance”. The area under the concentration time curve (AUC) was calculated by the linear/log trapezoidal rule. AUC_infinity_ values were extrapolated based on the last predicted concentration using the terminal elimination rate constant (lambda z). AUC_7h_ reflects the AUC in the first 7 h after dosage. Maximum concentration (C_max_) and time of maximum concentration (t_max_) are given as measured. The terminal half-life (t_1/2_) was calculated as ln(2)/lambda z. The volume of distribution based on the terminal phase (V_z_) was calculated as Dose/(lambda z × AUC_infinity_).

Heritability was estimated by structural equation modeling using the “mets” package for the statistical programming environment R [53, 54]. Variation in clearance was decomposed into additive genetic effects (A), dominant genetic effects (D), common environmental effects (C), and unique environmental effects (E). Fit of the models ACE, ADE, AE, and CE was tested by the Akaike-information criterion (AIC). In addition, the repeated administration of 50 mg talinolol to each subject was used to calculate the genetic component (rGC) as described by Kalow et al. [55, 56]. Variance within (Vw) the subjects and variance between (Vb) the subjects were calculated for all subjects who received 50 mg talinolol on three different occasions. The mean of 50 calculations of rGC is given. For each calculation of rGC only one randomly selected sibling of each pair was used, since the two siblings of each twin pair are not independent units.

The non-parametric Mann–Whitney U test was used for comparison of two groups and the Jonckheere–Terpstra test for comparison of more than two groups. In the analyses of the effects of genetic polymorphisms on talinolol clearance, an adjustment for multiple testing was done by using the Bonferroni correction method. Due to the high number of tests on genetic polymorphisms (ten in total), significance was determined if *p* < 0.005. Multiple linear regression analysis was performed for evaluating the effects of the genetic polymorphisms as well as the confounders sex, age, body mass index, consumption of protein, and consumption of vegetables. Protein and vegetable consumption was recorded using validated nutrition questionnaires [57] and calculated as the mean of items “meat”, “sausage”, “fish”, “dairy products”, and “eggs” and as the mean of items “salad and raw vegetable” and “cooked vegetable”, respectively.

## Results

Heritability of the variation in pharmacokinetics of talinolol was studied in 110 volunteers (42 monozygotic (MZ) and 13 dizygotic (DZ) twin pairs). No serious adverse events related to the study medication were reported. No statistically significant differences in the demographic data between MZ and DZ twin pairs were observed (Additional file 1).

Talinolol total clearance (Cl/F) measured after 50 mg talinolol varied 3.8-fold (in DZ) and 8.8-fold (in MZ); after adjustment for body weight, variation in clearance remained high with 2.9-fold and 6.3-fold in DZ and MZ twins, respectively. Similarly, strong variation was observed in AUC_infinity_ and AUC_7h_ (Table 1).

**Table 1:** Pharmacokinetic parameters of talinolol (50 mg dose)

		MZ twin pairs		DZ twin pairs	
Parameter	Unit	Mean ± SD	Median (Min–Max) Ratio Max/Min	Mean ± SD	Median (Min–Max) Ratio Max/Min
Clearance (Cl/F)	l/min	0.97 ± 0.42	0.87 (0.30–2.64) 8.8	0.82 ± 0.27	0.76 (0.37–1.39) 3.8
Clearance per body weight (Cl/F/kg)	ml/min/kg	14.4 ± 5.51	13.5 (6.16–38.5) 6.3	12.1 ± 3.8	11.6 (6.96–19.9) 2.9
AUC_infinity_	mg*min/l	62.8 ± 25.2	60.2 (22.8–171.1) 7.5	71.3 ± 23.8	68.6 (37.6–135.8) 3.6
AUC_7h_	mg*min/l	20.1 ± 9.10	19.3 (7.36–62.7) 8.5	24.9 ± 9.93	23.6 (11.7–54.8) 4.7
Central volume of distribution (V_z_)	l	1115 ± 510	1006 (293–2549) 8.7	889 ± 449	747 (302–2169) 7.2
Maximum plasma concentration (C_max_)	μg/l	94.3 ± 47.0	84.9 (23.3–258) 11.1	124 ± 55.4	115 (51.9–269) 5.2
Time of maximum plasma concentration (t_max_)	h	2.6 ± 0.7	2.6 (1.2–4.1) 3.4	2.6 ± 0.8	2.7 (1.0–3.9) 3.9
Terminal elimination half life (t_1/2_)	h	13.3 ± 4.0	12.2 (7.9–30.0) 3.8	11.9 ± 2.7	11.5 (7.2–18.1) 4.3

*MZ* monozygotic, *DZ* dizygotic, *SD* standard deviation. All pharmacokinetic parameters were calculated with the mean of up to three study days for each subject

**Fig. 1:**
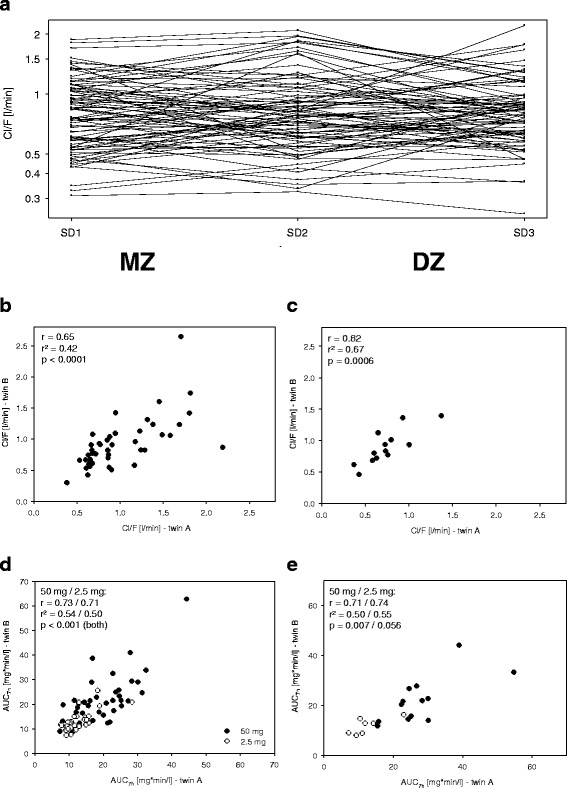
**a** Variation in Cl/F for the subjects who performed all three study days (*SD*) of the 50 mg talinolol study phase. **b**, **c** Correlation of the Cl/F after administration of 50 mg talinolol for all siblings from MZ (n = 42) and DZ (n = 13) twin pairs. **d**, **e** Correlation of the AUC_7h_ after administration of 50 mg talinolol (*filled circles*) and the dose-adjusted AUC_7h_ after administration of 2.5 mg talinolol (*empty circles*) for all analyzed twins. For **b**–**d** the Pearson correlation coefficients, the coefficients of determination, and the *p*-values are given. *MZ* monozygotic twin pairs, *DZ* dizygotic twin pairs, *Cl/F* total plasma clearance/bioavailability, *AUC*_*7h*_ area under the curve in the first 7 h after application, *SD* study day

No sequence effects were observed, i.e., the mean pharmacokinetic parameters were not significantly different between the study days. Clearance varied within subjects up to threefold between the three different study days (Fig. 1a) and the between-subject coefficient of variation (CV) was about 42 % for the whole study population. Within-twin pair correlations of the clearance were not significantly different between DZ and MZ twin pairs. With Pearson’s correlation coefficients of 0.65 (95 % confidence interval (CI) 0.43–0.80) for MZ and 0.82 (95 % CI 0.49–0.94) for DZ correlation, the correlation was unexpectedly nominally even higher in DZ than in MZ twin pairs (Fig. 1b, c). This indicates that common environmental effects substantially influenced the variation of the clearance.

According to structural equation modeling, common environmental (C) and unique environmental effects (E) best explained the variation in the clearance of talinolol (Table 2). Common environmental effects seem to account for 53.5 % and unique environmental effects for a further 46.5 % of the variation in the clearance (Table 2).

**Table 2:** Heritability of talinolol clearance (50 mg dose) according to structural equation modeling

Model	A	D	C	E	Chi2	*p*	AIC
	(95 % CI)	(95 % CI)	(95 % CI)	(95 % CI)			
Saturated	-	-	-	-	-	-	63.67
ACE	0.00 (0.00–0.00)	-	0.535 (0.346–0.724)	0.465 (0.276–0.654)	36.9	0.001	70.60
ADE	0.500 (0.282–0.717)	0.00 (0.00–0.00)	-	0.500 (0.282–0.717)	39.6	<0.001	73.30
AE	0.500 (0.282–0.717)	-	-	0.500 (0.282–0.717)	39.6	<0.001	71.30
**CE**	**-**	**-**	**0.535** **(0.346–0.724)**	**0.465** **(0.276–0.654)**	**37.9**	**0.002**	**68.60**

*A* additive genetic effects, *D* dominant genetic effects, *C* common environmental effects, *E* unique environmental effects, *95 % CI* 95 % confidence interval, *Chi2* chi-square value, *AIC* Akaike information criterion. *P* values were calculated with respect to the saturated model. The best fitting model (*CE*, *bold text*) was chosen based on the lowest AIC

An alternative model (AE) explained 50 % of the variation by additive genetic effects and 50 % by environmental effects. However, statistically this model was clearly inferior according to the AIC.

The pharmacokinetics of drugs usually depends on body weight and body weight is partially also genetically determined. Results were similar after adjustment of talinolol clearance for body weight with slightly more unique environmental effects (C = 45 % (95 % CI 24–66 %), E = 55 % (95 % CI 34–76 %)).

An alternative indicator for heritability can be estimated from the comparison of within- and between-subject variation. Based on the within- and between-subject variance with the three times repetition of 50 mg talinolol, the genetic component (rGC) was 0.38 (95 % CI 0.35–0.41) for talinolol total oral clearance and 0.53 (95 % CI 0.50–0.57) for talinolol AUC (Table 3). Within- and between-subject variance was similar for t_max_ and C_max_, which indicates there were no relevant genetic influences on these two parameters (Table 3).

**Table 3:** Heritability of talinolol pharmacokinetics (50 mg dose) according to the genetic component (rGC)

	Vb	Vw	rGC (95 % CI)
Cl/F	0.100	0.062	0.38 (0.35–0.41)
AUC	476.9	225.2	0.53 (0.50–0.57)
C_max_	1617	1671	
t_max_	0.50	0.63	

*rGC* genetic component, *Cl/F* total plasma clearance/bioavailability, *AUC* area under the curve, *C*_*max*_ maximum plasma concentration, *t*_*max*_ time of maximum plasma concentration, *Vb* variance between the subjects, *Vw* variance within each subject, *95 % CI* 95 % confidence interval. The genetic component was calculated as rGC = (Vb − Vw)/Vb as proposed by Kalow [52, 53].

Although our study indicated only low to moderate heritability in talinolol pharmacokinetic variation, there may be up to 38 % heritability in the clearance according to the repeated measurement approach. Therefore, we evaluated the influence of genetic polymorphisms on the efflux transporters MDR1, MDR5, MRP2, and BCRP as well as the most relevant polymorphisms in the influx transporters OCT1 and OATP1B1 on talinolol clearance. Considering the moderate sample size of our study, only those polymorphisms which have been associated with transport activity in earlier studies were analyzed. Heterozygous or homozygous carriers of *MDR1* 1236 T alleles and *MDR1* 2677 T alleles showed a significant increase (*p* = 0.0025 and 0.0015, respectively) in the total oral clearance of talinolol (Table 4). Clearance was increased by 44 and 48 % in homozygous carriers of *MDR1* 1236 T and 2677 T alleles compared to homozygous carriers of 1236C or 2677G alleles, respectively. All other polymorphisms in the genes coding for the efflux transporters MDR1, MRP2, MDR5, and BCRP were not significant predictors of talinolol total oral clearance (Table 4). Concerning the influx transporters OATP1B1 and OCT1, after adjustment for multiple testing, none of the variants known to be functional showed any significant association with talinolol clearance (Additional file 2).

**Table 4:** Influence of genetic polymorphisms in genes coding for efflux transporters on talinolol clearance

Gene	Genetic polymorphism	Variant	Genotype frequency (n) [%]	Clearance [l/min]
*MDR1*	C1236T	C/C	31 (34)	0.77 ± 0.28
	rs1128503	C/T	47 (52)	0.96 ± 0.37^a^
		T/T	21 (24)	1.11 ± 0.50^a^
	G2677T/A	G/G	29 (32)	0.77 ± 0.29
	(Ala893Ser/Thr)	G/T	47 (52)	0.96 ± 0.37^b^
	rs2032582	T/T	20 (22)	1.14 ± 0.50^b^
		G/A	2 (2)	0.74 ± 0.03
		A/A	0 (-)	-
		T/A	2 (2)	0.75 ± 0.10
	C3435T	C/C	19 (21)	0.83 ± 0.28
	rs1045642	C/T	62 (68)	0.97 ± 0.44
		T/T	19 (21)	0.92 ± 0.30
*MDR5*	C392T (Thr131Ile)	C/C	89 (98)	0.92 ± 0.37
	rs17143212	C/T	11 (12)	1.07 ± 0.54
		T/T	0 (-)	-
*MRP2*	-C24T	C/C	73 (80)	0.92 ± 0.39
	rs717620	C/T	21 (23)	1.03 ± 0.41
		T/T	6 (7)	0.73 ± 0.33
	G1249A (Val417Ile)	G/G	68 (75)	0.95 ± 0.41
	rs2273697	G/A	30 (33)	0.89 ± 0.37
		A/A	2 (2)	0.97 ± 0.05
*ABCG2*	G34A (Val12Met)	G/G	96 (105)	0.94 ± 0.40
	rs2231137	G/A	4 (5)	0.84 ± 0.25
		A/A	0 (-)	-
	C421A (Gln141Lys)	C/C	75 (82)	0.94 ± 0.37
	rs2231142	C/A	25 (28)	0.91 ± 0.46
		A/A	0 (-)	-

Data are given in mean ± standard deviation. ^a^There were significant differences between heterozygous (C/T) and homozygous (T/T) carriers of the *MDR1* 1236 T allele and homozygous carriers of the 1236C allele (C/C) as tested with the Jonckheere–Terpstra test (*p* = 0.0025). ^b^There were significant differences between heterozygous (G/T) and homozygous (T/T) carriers of the *MDR1* 2677 T allele and homozygous carriers of the 2677G allele (G/G) as tested with the Jonckheere–Terpstra test (*p* = 0.0015). Significance was determined after correction for multiple testing for *p* < 0.005 (Bonferroni correction)

Multiple linear regression analysis was performed to evaluate the possible influences of demographic and environmental factors potentially modulating the in vivo activity of drug membrane transporters (Table 5). In this multifactorial analysis none of the genetic polymorphisms remained significant, but sex, body mass index, protein, and vegetable consumption significantly explained variation in the talinolol clearance; these factors each explained only about 4 % of the total oral clearance of talinolol.

**Table 5:** Multiple linear regression analysis of talinolol (50 mg) clearance

Factor	r (r^2^)^a^	Coefficient	*p*
All factors	0.55 (0.30)	-	<0.001
Sex	0.20 (0.04)	0.184	0.044
Age	0.02 (<0.01)	0.001	NS
Body mass index	0.20 (0.04)	0.022	0.041
Protein consumption	−0.20 (0.04)	−0.144	0.046
Vegetable consumption	−0.20 (0.04)	−0.114	0.050
MDR1 C1236T	−0.05 (<0.01)	−0.039	NS
MDR1 G2677T/A	−0.03 (<0.01)	−0.020	NS

*NS* not significant. ^a^The correlation is given for the total model with all factors. The r^2^ (coefficient of determination) may indicate the fraction of the total variation explained by the respective factors

In addition to the 50 mg study, a low-dose study using 2.5 mg talinolol was performed. This study included 29 MZ and 7 DZ twin pairs. To enhance the feasibility of phenotyping, we restricted blood sampling to a shorter interval and therefore AUC_7h_ was the primary parameter. The median of the AUC_7h_ was 0.58 mg*min/l and varied between 0.36 and 1.42 mg*min/l. Between-twin pairs Pearson’s correlation coefficients of the AUC_7h_ were similar for both the 50-mg and the 2.5-mg groups (for 50 mg MZ = 0.73, DZ = 0.71; for 2.5 mg MZ = 0.71, DZ = 0.74; Fig. 1d, e). Structural equation modeling revealed that variation of the AUC_7h_ was best explained by common environmental effects (68.5 %, 95 % CI 51.1–85.8 %) and unique environmental effects (31.5 %, 95 % CI 14.2–48.9 %).

**Fig. 2:**
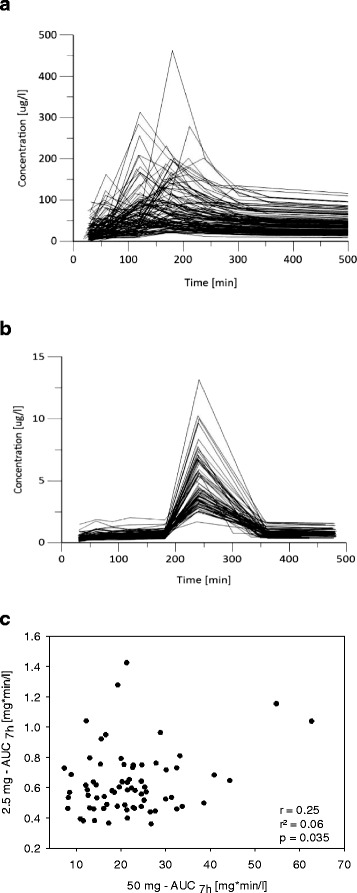
**a, b** Concentration time curves of one study day of the 50 mg talinolol study phase (**a**) and the 2.5 mg talinolol study phase (**b**). **c** Correlation of the AUC_7h_ after the administration of 50 mg and 2.5 mg talinolol. Shown are all 72 individuals who received both dosages. *AUC*_*7h*_ area under the curve in the first 7 hours after application

While significant inter-individual variation in the time of maximum blood concentration was observed after administration of 50 mg talinolol (Fig. 2a), variation in the time–concentration curves was apparently much lower in the 2.5 mg group (Fig. 2b). After 50 mg talinolol, the time of maximum plasma concentration (t_max_) varied between 1 and 4.1 h whereas after 2.5 mg, t_max_ was 4 h after administration in all 72 subjects (Fig. 2a, b). The maximum plasma concentration (C_max_) after 2.5 mg talinolol administration was 3.94 μg/l (median) and varied between 1.67 and 13.2 μg/l. Accordingly, the dose adjusted C_max_ was 1.57 μg/l/mg in the 2.5-mg study phase and 1.85 μg/l/mg in the 50-mg study phase.

The AUC_7h_ for the 50-mg and the 2.5-mg study phases showed a significant (*p* = 0.035) but only weak correlation, with a Person’s correlation coefficient of 0.25 (95 % CI 0.18–0.45) (Fig. 2c). Thus, according to the coefficient of determination, only about 6 % of the variation observed after administration of either dose was predictive for the variation in the other dose.

## Discussion

The impact of genetic variation on the activities of MDR1 and MRP2 has been extensively studied in the past 20 years but is still controversial [10, 11]. Conflicting results have been reported, particularly for the abundant and widely studied variants in *MDR1* (C1236T, G2677T/A, and C3435T) as well as for the -C24T and G1249A variants in *MRP2* [10]. Also, the genotype–phenotype correlations for many other frequent variants have mostly not been consistently reproduced, whereas those variants with strong penetrance and clear genotype–phenotype correlations are relatively rare. In this situation, we went back in the logical sequence of research to the important first question, namely, how much of the variation in drug membrane transport as tested with in vivo probe drugs is caused by heritable factors? One approach to answer this question and to differentiate between genetic and environmental factors is the use of twin studies. The results of twin studies may provide a unique basis to direct further research [58]. If a major part of the variation in the pharmacokinetics of probe drugs can be explained by genetic factors, then further approaches like whole-genome sequencing to search for the underlying factors may be promising. If results from twin studies reveal no or only moderate genetic effects, then such genome-wide screening might not be very promising or could require major effort to achieve adequate statistical power.

We chose talinolol as a probe drug because it is not metabolized to a relevant extent and in vitro and in vivo studies revealed that it may be a good probe drug for MDR1 as well as probably for MRP2 [37]. Generally, a probe drug has great heuristic potential allowing extrapolation from effects in one drug to numerous other drugs. However, this probe drug approach has limitations because in reality pharmacokinetics of every probe drug is not dependent on just one transporter or enzyme but probably on several, and many transporters or enzymes have multiple substrate binding sites with differential substrate affinities. With talinolol, we do not know quantitatively how much of the AUC or total clearance depends on MDR1 since data obtained in the MDR1 knockout rat [40] may be compromised by unspecific or secondary effects of the knockout; also, other factors limit extrapolation from rat to human.

More than usual with pharmacokinetic data, the mean pharmacokinetic parameters of talinolol differed between the studies. Its mean oral clearance was 0.93 l/min in our study and varied between 0.58 l/min [59] and 1.95 l/min (calculated from dose divided by given AUC_infinity_) [45] in other studies. Although this variation may result from minor differences in the pharmaceutical preparations, data analysis, and study populations, it could also indicate that some not yet clearly defined environmental factors have a major influence on the pharmacokinetics of talinolol.

To the best of our knowledge this is the first twin study on the heritability of talinolol pharmacokinetics as a marker of variation in drug membrane transport activities. We observed unexpectedly small heritability. The correlations between the siblings were nominally even higher for DZ than for MZ twin pairs (Fig. 1b, c) and, consistent with this, structural equation modeling revealed that common and unique environmental effects best explained the variation in talinolol oral clearance. Mechanistically, unique environmental effects include everything that acted randomly and not in the same manner in both siblings, whereas common environmental effects may include everything that is common to the siblings arising during pregnancy, childhood, and later life. Such common environmental effects may include all types of epigenetic effects but also any other factors which may be common due to growing up in the same family and environment.

The twin-study independent approach of Kalow et al. [55, 56], which uses repeated drug administration, revealed a low genetic component (rGC) of 0.38 and is generally in good agreement with the result from structural equation modeling. However, this component of 38 % includes both genetic effects and common environmental effects. This means that less (probably even much less) than 38 % of the variation is due to heritable factors. In contrast to the low genetic component in this study, other studies reveal high genetic components for other pharmacological variability, e.g., between 0.95 and 0.98 for the activity of N-acetyltransferase 2 (NAT2) [52, 55], 0.96 for plasma clearance of midazolam [60], 0.94 for the ethinylestradiol serum AUC [60], and 0.91 for the plasma AUC of metoprolol [51].

Several genetic polymorphisms in drug membrane transporters have already been analyzed in relation to talinolol pharmacokinetics. In our study, the genetic variants of *MDR1* C1236T and G2677T/A were significantly associated with talinolol oral clearance, but this association was weak and even not significant after including other factors in a multifactorial analysis. For both polymorphisms clearance was enhanced in subjects heterozygously or homozygously carrying the 1236 T and 2677 T alleles, indicating higher activity of MDR1 compared to homozygous carriers of the 1236C or 2677G alleles. In line with this, both polymorphisms showed a lower AUC in subjects carrying hetero- or homozygous variants (1236 T and 2677 T or A) in another independent study on the oral bioavailability of talinolol [44]. However, two further studies found no significant effects of C3435T and G2677T/A [45, 61] as well as C1236T [45] on the talinolol AUC, indicating only weak functional effects and/or big effects of other not yet fully understood factors.

In this study we did not observe effects of some frequently studied and apparently functional *MRP2*, MDR5, and ABCG2 polymorphisms on talinolol clearance. The MRP2 finding is in contrast to Haenisch et al. [47], who reported an association of a lower AUC (and a lower oral bioavailability) and the 1249A variant. However, confirmation of earlier genotype–phenotype associations was not the primary aim of the present study and would have required a substantially bigger sample size, particularly for the quantitatively moderate effects.

The effects of genetic variation in efflux transporters on talinolol pharmacokinetics may be complicated by additional influences of genetically polymorphic influx transporters. A study by Bernsdorf et al. reported a significantly decreased talinolol half-life in carriers of the *1b variant in organic anion transporter *OATP1B1* [48]. The *1b variant seems to code for a protein with high activity [62] but, in the study by Bernsdorf et al., subjects carrying the low-activity allele *15 of *OATP1B1* also showed a decreased half-life. In line with these results, our study revealed that heterozygous carriers of the *1b allele (n = 21) tended to have a higher clearance compared to carriers of the wild-type allele *1a (Additional file 2) but homozygous carriers of the *1b allele (n = 4) had a lower clearance. The lack of consistency and the lack of a consistent effect in our study of the low activity allele *5 do not support a relevant role for OATP1B1 in the pharmacokinetics of talinolol.

Talinolol is a relatively hydrophilic drug and with a pKa of 9.3, about 98 % of the molecules are positively charged at physiological pH [50]. It is therefore a possible substrate of organic cation transporters [44]. For this reason we studied the influence of genetic variants in the genetically highly polymorphic transporter *OCT1* on talinolol clearance. Surprisingly, although not significant, talinolol oral clearance tended to be increased in carriers of one or two reduced activity *OCT1* alleles. Further in vitro research is required to elucidate whether OATP1B1 or OCT1 may enhance cellular uptake of talinolol.

Interestingly, protein and vegetable consumption both statistically significantly increased talinolol clearance. Our study was not designed to elucidate the underlying substances and mechanisms but it is reasonable that the pharmacokinetics of the mostly not metabolized drug talinolol is strongly dependent on transport activities in the intestinal mucosa and expression in these cells should depend on diet.

In an additional study, a subgroup of the volunteers received a low oral dose of 2.5 mg talinolol. The aim of this low-dose study was to enable a comparison between it and the 50 mg study. The so-called microdosing design has repeatedly been shown to be a valuable tool in the drug development process [63]. Although microdosing is defined by administration of doses below 100 μg, and in this sense we did not perform microdosing, the observations with our *low range millidosing* are notable. Interestingly, we observed quite different results compared to the therapeutic dose. The dose-normalized AUC_7h_ was only about 50 % in the low dose talinolol group compared with the AUC from the comparable time interval in the 50 mg talinolol group. Also, t_max_ was achieved significantly earlier in the 50 mg talinolol group. These differences were already noted by Wetterich et al. [50]. One explanation may be that, in the high dose group, efflux transporters like MDR1 may be saturated very early so drug elimination, especially due to intestinal efflux, is delayed [50]. The very low correlation of the AUC_7h_ for 2.5 mg and 50 mg talinolol (Fig. 2c) may similarly indicate first that this may be one example of a substance for which microdosing is not highly predictive for pharmacokinetics in therapeutic doses and second that the relevant transport processes and proteins involved may be different depending on the doses. This is supported by the fact that this correlation (Fig. 2c) was much lower than the correlation between the different study days with the same dose and between the siblings (Fig. 1d, e). In contrast to our study, another study with another MDR1 probe drug, fexofenadine, showed more rapid absorption of the drug with the smaller dose [64]. In this other study, however, 100 μg of fexofenadine was administered in 10 ml physiological saline and quickly swallowed and the high dose of 120 mg was administered as a tablet. Therefore, the different formulations may explain the contradictory results to our study.

## Conclusions

The absorption, distribution, and elimination of talinolol seem to be mainly determined by environmental effects. This may indicate that the in vivo activity of MDR1 may also be dominantly determined by environmental factors. Some data presented in this study, however, may raise doubts about whether or not talinolol is really a good in vivo probe drug for MDR1. Further experimental and clinical research on the role of OATP1B1 and OCT1 may be scientifically interesting, but from our data one might simply conclude that frequent genetic polymorphisms with dominant effects on talinolol pharmacokinetics do not exist.

## Additional files

Additional file 1: Demographic data of the studied individuals. (DOCX 15 kb)
Additional file 2: Influence of OCT1 and OATP1B1 on talinolol clearance. (DOCX 15 kb)

## Abbreviations

ABCC2: ATP binding cassette subfamily C member 2
AIC: Akaike information criterion
AUC: Area under the curve
Cl/F: Total plasma clearance/bioavailability
CV: Coefficient of variation
DZ: Dizygotic
LC-MS/MS: Liquid chromatography–tandem mass spectrometry
LOQ: Limit of quantification
MDR1: Multi-drug resistance protein 1 (P-glycoprotein)
MRP2: Multidrug resistance-associated protein 2
MZ: Monozygotic
OATP1B1: Organic anion-transporting polypeptide 1B1
OCT1: Organic cation transporter 1
rGC: Genetic component

## References

[1] Pastan I, Gottesman MM (1991). Multidrug resistance. Annu Rev Med.

[2] Norris MD, Bordow SB, Marshall GM, Haber PS, Cohn SL, Haber M (1996). Expression of the gene for multidrug-resistance-associated protein and outcome in patients with neuroblastoma. N Engl J Med.

[3] Juliano RL, Ling V (1976). A surface glycoprotein modulating drug permeability in Chinese hamster ovary cell mutants. Biochim Biophys Acta.

[4] Chen CJ, Chin JE, Ueda K, Clark DP, Pastan I, Gottesman MM, Roninson IB (1986). Internal duplication and homology with bacterial transport proteins in the mdr1 (P-glycoprotein) gene from multidrug-resistant human cells. Cell.

[5] Schinkel AH (1997). The physiological function of drug-transporting P-glycoproteins. Semin Cancer Biol.

[6] Greiner B, Eichelbaum M, Fritz P, Kreichgauer HP, von Richter O, Zundler J, Kroemer HK (1999). The role of intestinal P-glycoprotein in the interaction of digoxin and rifampin. J Clin Invest.

[7] Fromm MF (2000). P-glycoprotein: a defense mechanism limiting oral bioavailability and CNS accumulation of drugs. Int J Clin Pharmacol Ther.

[8] Fellay J, Marzolini C, Meaden ER, Back DJ, Buclin T, Chave JP, Decosterd LA, Furrer H, Opravil M, Pantaleo G (2002). Response to antiretroviral treatment in HIV-1-infected individuals with allelic variants of the multidrug resistance transporter 1: a pharmacogenetics study. Lancet.

[9] Sadhasivam S, Chidambaran V, Zhang X, Meller J, Esslinger H, Zhang K, Martin LJ, McAuliffe J (2015). Opioid-induced respiratory depression: ABCB1 transporter pharmacogenetics. Pharmacogenomics J.

[10] Bruhn O, Cascorbi I (2014). Polymorphisms of the drug transporters ABCB1, ABCG2, ABCC2 and ABCC3 and their impact on drug bioavailability and clinical relevance. Expert Opin Drug Metab Toxicol.

[11] Wolking S, Schaeffeler E, Lerche H, Schwab M, Nies AT (2015). Impact of genetic polymorphisms of ABCB1 (MDR1, P-glycoprotein) on drug disposition and potential clinical implications: update of the literature. Clin Pharmacokinet.

[12] Geick A, Eichelbaum M, Burk O (2001). Nuclear receptor response elements mediate induction of intestinal MDR1 by rifampin. J Biol Chem.

[13] Slater LM, Sweet P, Stupecky M, Gupta S (1986). Cyclosporine-A reverses vincristine and daunorubicin resistance in acute lymphatic-leukemia in vitro. J Clin Investig.

[14] Tsuruo T, Iida H, Tsukagoshi S, Sakurai Y (1981). Overcoming of vincristine resistance in P388 leukemia in vivo and in vitro through enhanced cytotoxicity of vincristine and vinblastine by verapamil. Cancer Res.

[15] Hoffmeyer S, Burk O, von Richter O, Arnold HP, Brockmoller J, Johne A, Cascorbi I, Gerloff T, Roots I, Eichelbaum M, Brinkmann U (2000). Functional polymorphisms of the human multidrug-resistance gene: multiple sequence variations and correlation of one allele with P-glycoprotein expression and activity in vivo. Proc Natl Acad Sci U S A.

[16] Sakaeda T, Nakamura T, Horinouchi M, Kakumoto M, Ohmoto N, Sakai T, Morita Y, Tamura T, Aoyama N, Hirai M (2001). MDR1 genotype-related pharmacokinetics of digoxin after single oral administration in healthy Japanese subjects. Pharm Res.

[17] Becquemont L, Verstuyft C, Kerb R, Brinkmann U, Lebot M, Jaillon P, Funck-Brentano C (2001). Effect of grapefruit juice on digoxin pharmacokinetics in humans. Clin Pharmacol Ther.

[18] von Ahsen N, Richter M, Grupp C, Ringe B, Oellerich M, Armstrong VW (2001). No influence of the MDR-1 C3435T polymorphism or a CYP3A4 promoter polymorphism (CYP3A4-V allele) on dose-adjusted cyclosporin A trough concentrations or rejection incidence in stable renal transplant recipients. Clin Chem.

[19] Min DI, Ellingrod VL (2002). C3435T mutation in exon 26 of the human MDR1 gene and cyclosporine pharmacokinetics in healthy subjects. Ther Drug Monit.

[20] Drescher S, Schaeffeler E, Hitzl M, Hofmann U, Schwab M, Brinkmann U, Eichelbaum M, Fromm MF (2002). MDR1 gene polymorphisms and disposition of the P-glycoprotein substrate fexofenadine. Br J Clin Pharmacol.

[21] Verstuyft C, Schwab M, Schaeffeler E, Kerb R, Brinkmann U, Jaillon P, Funck-Brentano C, Becquemont L (2003). Digoxin pharmacokinetics and MDR1 genetic polymorphisms. Eur J Clin Pharmacol.

[22] Wang D, Sadee W (2006). Searching for polymorphisms that affect gene expression and mRNA processing: example ABCB1 (MDR1). AAPS J.

[23] Kimchi-Sarfaty C, Oh JM, Kim IW, Sauna ZE, Calcagno AM, Ambudkar SV, Gottesman MM (2007). A “silent” polymorphism in the MDR1 gene changes substrate specificity. Science.

[24] Fung KL, Gottesman MM (2009). A synonymous polymorphism in a common MDR1 (ABCB1) haplotype shapes protein function. Biochim Biophys Acta.

[25] Kim RB, Leake BF, Choo EF, Dresser GK, Kubba SV, Schwarz UI, Taylor A, Xie HG, McKinsey J, Zhou S (2001). Identification of functionally variant MDR1 alleles among European Americans and African Americans. Clin Pharmacol Ther.

[26] Kroetz DL, Pauli-Magnus C, Hodges LM, Huang CC, Kawamoto M, Johns SJ, Stryke D, Ferrin TE, DeYoung J, Taylor T (2003). Sequence diversity and haplotype structure in the human ABCB1 (MDR1, multidrug resistance transporter) gene. Pharmacogenetics.

[27] Cui Y, Konig J, Buchholz JK, Spring H, Leier I, Keppler D (1999). Drug resistance and ATP-dependent conjugate transport mediated by the apical multidrug resistance protein, MRP2, permanently expressed in human and canine cells. Mol Pharmacol.

[28] Schaub TP, Kartenbeck J, Konig J, Spring H, Dorsam J, Staehler G, Storkel S, Thon WF, Keppler D (1999). Expression of the MRP2 gene-encoded conjugate export pump in human kidney proximal tubules and in renal cell carcinoma. J Am Soc Nephrol.

[29] Kamisako T, Gabazza EC, Ishihara T, Adachi Y (1999). Molecular aspects of organic compound transport across the plasma membrane of hepatocytes. J Gastroenterol Hepatol.

[30] Keppler D (2014). The roles of MRP2, MRP3, OATP1B1, and OATP1B3 in conjugated hyperbilirubinemia. Drug Metab Dispos.

[31] Pedersen JM, Matsson P, Bergstrom CA, Norinder U, Hoogstraate J, Artursson P (2008). Prediction and identification of drug interactions with the human ATP-binding cassette transporter multidrug-resistance associated protein 2 (MRP2; ABCC2). J Med Chem.

[32] Naesens M, Kuypers DR, Verbeke K, Vanrenterghem Y (2006). Multidrug resistance protein 2 genetic polymorphisms influence mycophenolic acid exposure in renal allograft recipients. Transplantation.

[33] Levesque E, Benoit-Biancamano MO, Delage R, Couture F, Guillemette C (2008). Pharmacokinetics of mycophenolate mofetil and its glucuronide metabolites in healthy volunteers. Pharmacogenomics.

[34] Ogasawara K, Chitnis SD, Gohh RY, Christians U, Akhlaghi F (2013). Multidrug resistance-associated protein 2 (MRP2/ABCC2) haplotypes significantly affect the pharmacokinetics of tacrolimus in kidney transplant recipients. Clin Pharmacokinet.

[35] Renders L, Frisman M, Ufer M, Mosyagin I, Haenisch S, Ott U, Caliebe A, Dechant M, Braun F, Kunzendorf U, Cascorbi I (2007). CYP3A5 genotype markedly influences the pharmacokinetics of tacrolimus and sirolimus in kidney transplant recipients. Clin Pharmacol Ther.

[36] FDA. Drug interaction studies--study design, data analysis, implications for dosing, and labeling recommendations. 2012. http://www.fda.gov/downloads/Drugs/GuidanceComplianceRegulatoryInformation/Guidances/UCM292362.pdf. 10.1038/sj.clpt.6100054 17259955

[37] Ma JD, Tsunoda SM, Bertino JS, Trivedi M, Beale KK, Nafziger AN (2010). Evaluation of in vivo P-glycoprotein phenotyping probes: a need for validation. Clin Pharmacokinet.

[38] Oertel R, Richter K, Trausch B, Berndt A, Gramatte T, Kirch W (1994). Elucidation of the structure of talinolol metabolites in man. Determination of talinolol and hydroxylated talinolol metabolites in urine and analysis of talinolol in serum. J Chromatogr B Biomed Appl.

[39] Trausch B, Oertel R, Richter K, Gramatte T (1995). Disposition and bioavailability of the beta 1-adrenoceptor antagonist talinolol in man. Biopharm Drug Dispos.

[40] Chu X, Zhang Z, Yabut J, Horwitz S, Levorse J, Li XQ, Zhu L, Lederman H, Ortiga R, Strauss J (2012). Characterization of multidrug resistance 1a/P-glycoprotein knockout rats generated by zinc finger nucleases. Mol Pharmacol.

[41] Gramatte T, Oertel R (1999). Intestinal secretion of intravenous talinolol is inhibited by luminal R-verapamil. Clin Pharmacol Ther.

[42] Giessmann T, May K, Modess C, Wegner D, Hecker U, Zschiesche M, Dazert P, Grube M, Schroeder E, Warzok R (2004). Carbamazepine regulates intestinal P-glycoprotein and multidrug resistance protein MRP2 and influences disposition of talinolol in humans. Clin Pharmacol Ther.

[43] Westphal K, Weinbrenner A, Zschiesche M, Franke G, Knoke M, Oertel R, Fritz P, von Richter O, Warzok R, Hachenberg T (2000). Induction of P-glycoprotein by rifampin increases intestinal secretion of talinolol in human beings: a new type of drug/drug interaction. Clin Pharmacol Ther.

[44] Schwarz UI, Hanso H, Oertel R, Miehlke S, Kuhlisch E, Glaeser H, Hitzl M, Dresser GK, Kim RB, Kirch W (2007). Induction of intestinal P-glycoprotein by St John’s wort reduces the oral bioavailability of talinolol. Clin Pharmacol Ther.

[45] Schwarz UI, Seemann D, Oertel R, Miehlke S, Kuhlisch E, Fromm MF, Kim RB, Bailey DG, Kirch W (2005). Grapefruit juice ingestion significantly reduces talinolol bioavailability. Clin Pharmacol Ther.

[46] Schwarz UI, Gramatte T, Krappweis J, Berndt A, Oertel R, von Richter O, Kirch W (1999). Unexpected effect of verapamil on oral bioavailability of the beta-blocker talinolol in humans. Clin Pharmacol Ther.

[47] Haenisch S, May K, Wegner D, Caliebe A, Cascorbi I, Siegmund W (2008). Influence of genetic polymorphisms on intestinal expression and rifampicin-type induction of ABCC2 and on bioavailability of talinolol. Pharmacogenet Genomics.

[48] Bernsdorf A, Giessmann T, Modess C, Wegner D, Igelbrink S, Hecker U, Haenisch S, Cascorbi I, Terhaag B, Siegmund W (2006). Simvastatin does not influence the intestinal P-glycoprotein and MPR2, and the disposition of talinolol after chronic medication in healthy subjects genotyped for the ABCB1, ABCC2 and SLCO1B1 polymorphisms. Br J Clin Pharmacol.

[49] Zheng M, Zhang H, Dill DL, Clark JD, Tu S, Yablonovitch AL, Tan MH, Zhang R, Rujescu D, Wu M (2015). The role of Abcb5 alleles in susceptibility to haloperidol-induced toxicity in mice and humans. PLoS Med.

[50] Wetterich U, Spahn-Langguth H, Mutschler E, Terhaag B, Rosch W, Langguth P (1996). Evidence for intestinal secretion as an additional clearance pathway of talinolol enantiomers: concentration- and dose-dependent absorption in vitro and in vivo. Pharm Res.

[51] Matthaei J, Brockmoller J, Tzvetkov MV, Sehrt D, Sachse-Seeboth C, Hjelmborg JB, Moller S, Halekoh U, Hofmann U, Schwab M, Kerb R (2015). Heritability of metoprolol and torsemide pharmacokinetics. Clin Pharmacol Ther.

[52] Matthaei J, Tzvetkov MV, Strube J, Sehrt D, Sachse-Seeboth C, Hjelmborg JB, Moller S, Halekoh U, Hofmann U, Schwab M (2016). Heritability of caffeine metabolism: Environmental effects masking genetic effects on CYP1A2 activity but not on NAT2. Clin Pharmacol Ther.

[53] Holst K, Scheike T (2015). mets: analysis of multivariate event times, R package version 1.1.1.

[54] R Core Team. R: A language and environment for statistical computing. Vienna: R foundation for statistical computing; 2015. http://www.R-project.org.

[55] Kalow W, Tang BK, Endrenyi L (1998). Hypothesis: comparisons of inter- and intra-individual variations can substitute for twin studies in drug research. Pharmacogenetics.

[56] Kalow W, Endrenyi L, Tang B (1999). Repeat administration of drugs as a means to assess the genetic component in pharmacological variability. Pharmacology.

[57] Winkler G, Doring A (1998). Validation of a short qualitative food frequency list used in several German large scale surveys. Z Ernahrungswiss.

[58] Vesell ES. Twin studies in pharmacogenetics. Hum Genet Suppl. 1978;1:19–30. 10.1007/978-3-642-67179-1_4 285030

[59] Westphal K, Weinbrenner A, Giessmann T, Stuhr M, Franke G, Zschiesche M, Oertel R, Terhaag B, Kroemer HK, Siegmund W (2000). Oral bioavailability of digoxin is enhanced by talinolol: evidence for involvement of intestinal P-glycoprotein. Clin Pharmacol Ther.

[60] Ozdemir V, Kalow W, Tang BK, Paterson AD, Walker SE, Endrenyi L, Kashuba ADM (2000). Evaluation of the genetic component of variability in CYP3A4 activity: a repeated drug administration method. Pharmacogenetics.

[61] Siegmund W, Ludwig K, Giessmann T, Dazert P, Schroeder E, Sperker B, Warzok R, Kroemer HK, Cascorbi I (2002). The effects of the human MDR1 genotype on the expression of duodenal P-glycoprotein and disposition of the probe drug talinolol. Clin Pharmacol Ther.

[62] Mwinyi J, Johne A, Bauer S, Roots I, Gerloff T (2004). Evidence for inverse effects of OATP-C (SLC21A6) 5 and 1b haplotypes on pravastatin kinetics. Clin Pharmacol Ther.

[63] Burt T, Yoshida K, Lappin G, Vuong L, John C, de Wildt SN, Sugiyama Y, Rowland M. Microdosing and other phase-0 clinical trials: facilitating translation in drug development. Clin Transl Sci. 2016;9:74-88. 10.1111/cts.12390 PMC5351314 26918865

[64] Lappin G, Shishikura Y, Jochemsen R, Weaver RJ, Gesson C, Houston B, Oosterhuis B, Bjerrum OJ, Rowland M, Garner C (2010). Pharmacokinetics of fexofenadine: evaluation of a microdose and assessment of absolute oral bioavailability. Eur J Pharm Sci.

