# Supplementary material for: Low heritability in pharmacokinetics of talinolol: a pharmacogenetic twin study on the heritability of the pharmacokinetics of talinolol, a putative probe drug of MDR1 and other membrane transporters
Source: Genome Med. 2016 Nov 8;8:119. doi: 10.1186/s13073-016-0372-2 (PMC5101708; doi:10.1186/s13073-016-0372-2)
Supplement: Additional file 1: — Demographic data of the studied individuals. (DOCX 15 kb) [file 13073_2016_372_MOESM1_ESM.docx]

**Additional file 1** Demographic data of the studied individuals.

|  | Total | **Sex** | | **Age** | **Weight** | **Height** | **BMI** |
| --- | --- | --- | --- | --- | --- | --- | --- |
|  |  | female | male | [years] | [kg] | [m] | [kg/m^2^] |
|  | n | n | n | Mean | | | |
|  | (% of each group) | | | (Range) | | | |
| **Main study (50 mg talinolol)** | | | | | | | |
| Monozygotic | 84  (76) | 52  (62) | 32  (38) | 25.6  (18 - 56) | 66.9  (48 - 97.5) | 1.72  (1.55 - 1.95) | 22.6  (17.9 - 34.1) |
|  |  |  |  |  |  |  |  |
| Dizygotic | 26  (24) | 18  (69) | 8  (31) | 22.3  (18 - 36) | 67.7  (53.5 - 83.5) | 1.71  (1.60 - 1.95) | 23.2  (17.7 - 29.7) |
|  |  |  |  |  |  |  |  |
| **Add-on study (2.5 mg talinolol)** | | | | | | | |
| Monozygotic | 58  (81) | 40  (69) | 18  (31) | 25.3  (18 - 51) | 64.6  (48 - 97.5) | 1.70  (1.55 - 1.95) | 22.4  (17.9 - 34.1) |
|  |  |  |  |  |  |  |  |
| Dizygotic | 14  (19) | 12  (86) | 2  (14) | 21.9  (18 - 27) | 67.0  (53.5 - 83.5) | 1.70  (1.60 - 1.95) | 23.4  (18.9 - 29.7) |

BMI = body mass index.
