# Supplementary material for: Low heritability in pharmacokinetics of talinolol: a pharmacogenetic twin study on the heritability of the pharmacokinetics of talinolol, a putative probe drug of MDR1 and other membrane transporters
Source: Genome Med. 2016 Nov 8;8:119. doi: 10.1186/s13073-016-0372-2 (PMC5101708; doi:10.1186/s13073-016-0372-2)
Supplement: Additional file 2: — Influence of OCT1 and OATP1B1 on talinolol clearance. (DOCX 15 kb) [file 13073_2016_372_MOESM2_ESM.docx]

**Additional file 2**  Influence of *OCT1* and *OATP1B1* on talinolol clearance

| Gene | Genotype | Genotype frequency (n) [%] | Clearance [l/min] |
| --- | --- | --- | --- |
| *OCT1* | H1/H1 | 54 (60) | 0.87 ± 0.36 |
|  |  |  |  |
|  | H1/H2 | 14 (16) | 1.05 ± 0.40 |
|  | H1/H3 | 14 (16) | 1.01 ± 0.51 |
|  | H1/H4 | 2 (2) | 0.64 ± 0.05 |
|  | H1/H5 | 3 (3) | 0.59 ± 0.21 |
|  | All H1/Hx | 34 (37) | 0.98 ± 0.45 |
|  |  |  |  |
|  | H2/H2 | 2 (2) | 0.73 ± 0.06 |
|  | H2/H3 | 2 (2) | 0.81 ± 0.08 |
|  | H2/H5 | 4 (4) | 1.35 ± 0.21 |
|  | H3/H3 | 2 (2) | 1.32 ± 0.37 |
|  | H3/H4 | 1 (1) | 1.36 |
|  | H3/H5 | 1 (1) | 0.93 |
|  | H3/H10 | 1 (1) | 1.01 |
|  | All Hx/Hx | 12 (13) | 1.11 ± 0.32 |
| *OATP1B1* | *1a/*1a | 27 (30) | 0.89 ± 0.39 |
|  | *1a/*1b | 13 (14) | 1.30 ± 0.56 |
|  | *1a/*5 | 5 (5) | 1.08 ± 0.26 |
|  | *1a/*14 | 16 (18) | 0.89 ± 0.32 |
|  | *1a/*15 | 16 (18) | 0.85 ± 0.35 |
|  | *1b/*1b | 3 (4) | 0.67 ± 0.14 |
|  | *1b/*14 | 5 (5) | 1.02 ± 0.10 |
|  | *1b/*15 | 2 (2) | 0.77 ± 0.004 |
|  | *5/*14 | 2 (2) | 0.53 ± 0.15 |
|  | *14/*15 | 5 (5) | 0.94 ± 0.35 |
|  | *15/*15 | 6 (7) | 0.88 ± 0.32 |

Data is given in mean ± SD. Hx = OCT1 allele ≠ H1.
